# Antimicrobial effectiveness of intracanal medicaments against *Enterococcus faecalis* in endodontics: a systematic review with structured domain-based interpretative synthesis and exploratory meta-analysis

**DOI:** 10.3389/froh.2026.1854046

**Published:** 2026-05-29

**Authors:** Carlos M. Ardila, Eliana Pineda-Vélez, Anny Marcela Vivares-Builes

**Affiliations:** 1 Department of Periodontics, Saveetha Dental College, and Hospitals, Saveetha Institute of Medical and Technical Sciences, Chennai, India; 2 Basic Sciences Department, Biomedical Stomatology Research Group, Faculty of Dentistry, Universidad de Antioquia U de A, Medellín, Colombia

**Keywords:** anti-bacterial agents, calcium hydroxide, enterococcus faecalis, root canal therapy, tooth root

## Abstract

**Background:**

Persistent endodontic infections are frequently associated with *Enterococcus faecalis*, a pathogen capable of biofilm formation and dentinal tubule penetration, contributing to treatment failure.

**Objectives:**

This systematic review aimed to evaluate the antimicrobial effectiveness of intracanal medicaments against *E. faecalis*, compare different formulations, and explore patterns of bacterial resistance and tolerance using a structured domain-based interpretative synthesis and exploratory meta-analysis.

**Methods:**

A systematic review was conducted following PRISMA 2020 guidelines. Searches were performed in PubMed/MEDLINE, Scopus, and Embase. Eligible studies included *in vitro*, *ex vivo*, and clinical investigations reporting quantitative antimicrobial outcomes. A structured domain-based interpretative synthesis was performed across key domains, and meta-analysis was conducted when feasible.

**Results:**

Twelve studies were included. Antibiotic-based medicaments, particularly triple antibiotic paste, generally showed comparatively stronger antimicrobial performance, especially in studies assessing bacterial reduction and biofilm-related outcomes. Calcium hydroxide showed variable and generally lower efficacy, especially in deeper dentinal regions, though combination therapies improved performance. Novel agents, including nanoparticle-based systems and bioactive compounds, showed promising but heterogeneous results. Quantitative pooling was limited to two studies and revealed extreme heterogeneity with imprecise estimates. Overall certainty of evidence ranged from low to moderate.

**Conclusions:**

Intracanal medicaments exhibit consistent antimicrobial activity against *E. faecalis*, with comparatively stronger performance observed for antibiotic-based formulations in the available experimental evidence. However, substantial heterogeneity and limited high-quality clinical evidence constrain definitive conclusions. These findings suggest potential improvements in intracanal disinfection strategies, pending further standardized and clinically robust investigations.

**Systematic Review Registration:**

https://www.crd.york.ac.uk/PROSPERO/recorddashboard, identifier CRD420261345958.

## Introduction

Persistent endodontic infections remain a significant challenge in contemporary endodontics, often leading to treatment failure despite advances in instrumentation and irrigation protocols. Among the microorganisms implicated, *Enterococcus faecalis* has been consistently identified as a key pathogen associated with post-treatment apical periodontitis. This bacterium exhibits remarkable adaptability, including the ability to survive in nutrient-deprived environments, penetrate dentinal tubules, and form resilient biofilms, which collectively contribute to its persistence following root canal therapy (1, 2).

Intracanal medicaments are widely used as an adjunct to mechanical and chemical debridement with the aim of reducing microbial load between appointments. Calcium hydroxide (CH) has historically been the most commonly used intracanal medicament due to its high pH and antimicrobial properties. However, several studies have suggested that *E. faecalis* can resist or tolerate the effects of CH, particularly within dentinal tubules and biofilm structures (3, 4). This has led to the exploration of alternative or adjunctive medicaments, including chlorhexidine-based formulations, antibiotic pastes such as triple antibiotic paste (TAP), and more recently, nanoparticle-based and modified antimicrobial systems (5, 6).

The available scientific evidence evaluating the antimicrobial effectiveness of these intracanal medicaments is extensive but characterized by considerable heterogeneity. Differences in experimental models (*in vitro*, *ex vivo*, and clinical), bacterial inoculation protocols, medicament concentrations and vehicles, as well as outcome measures such as colony-forming units (CFU), logarithmic reduction, minimum inhibitory concentration (MIC), and minimum bactericidal concentration (MBC), make direct comparisons challenging. Furthermore, the complexity of *E. faecalis* behavior in biofilm and dentin environments introduces additional variability in reported outcomes (7, 8).

Despite the growing body of literature, there remains uncertainty regarding the relative effectiveness of different intracanal medicaments against *E. faecalis*, particularly in clinically relevant conditions that simulate dentinal tubule penetration and biofilm persistence. Moreover, there is a lack of structured synthesis that integrates quantitative microbiological outcomes while also interpreting patterns of bacterial resistance and tolerance. This gap limits the ability of clinicians and researchers to draw evidence-based conclusions regarding optimal intracanal disinfection strategies.

Therefore, a comprehensive and methodologically rigorous synthesis of the available evidence is warranted. The present systematic review is designed to address this gap by evaluating the antimicrobial effectiveness of intracanal medicaments against *E. faecalis* using standardized microbiological outcomes, including CFU counts, log reduction, MIC, and MBC. In addition, this review uses a structured domain-based interpretative synthesis to organize patterns of antimicrobial performance and potential resistance or tolerance mechanisms across different experimental contexts.

The primary objective of this systematic review is to assess the antimicrobial effectiveness of intracanal medicaments against *E. faecalis*. The secondary objectives are to compare the effectiveness across different medicaments and to explore potential patterns of bacterial resistance and tolerance in dentin and biofilm environments. By integrating quantitative evidence with functional interpretation, this review aims to provide a more comprehensive understanding of intracanal disinfection strategies and their clinical implications.

## Materials and methods

This systematic review with structured domain-based interpretative synthesis and exploratory meta-analysis was conducted in accordance with the Preferred Reporting Items for Systematic Reviews and Meta-Analyses (PRISMA 2020) guidelines (9). The protocol was developed *a priori* and registered in the International Prospective Register of Systematic Reviews (PROSPERO; CRD420261345958).

### Eligibility criteria

The eligibility criteria were defined using the PICOS framework, tailored to the specific objectives of this review.

#### Participants (P)

Studies evaluating *E. faecalis* as the primary microorganism in endodontic infection models, including dentin, biofilm, or clinical root canal systems.

#### Intervention (I)

Intracanal medicaments such as calcium hydroxide, chlorhexidine used as a medicament, TAP, modified TAP formulations, nanoparticle-based systems, and other alternative antimicrobial formulations.

#### Comparisons (C)

Comparisons between different intracanal medicaments, untreated controls, or vehicle controls.

#### Outcomes (O)

Quantitative microbiological outcomes, including CFU, logarithmic reduction, MIC, MBC, as well as indicators of antimicrobial persistence, resistance, or tolerance where reported.

#### Study design (S)

*In vitro*, *ex vivo*, and clinical studies reporting quantitative antimicrobial outcomes.

### Exclusion criteria

Studies were excluded if *E. faecalis* was not the primary microorganism or if results were not reported separately. Additionally, studies focusing exclusively on irrigants, activation techniques, sealers, photodynamic therapy, reviews, case reports, and studies lacking quantitative data were excluded.

### Information sources and search strategy

A comprehensive search strategy was implemented across three electronic databases: PubMed/MEDLINE, Scopus, and Embase (via Ovid). The search included studies published up to February 2026, without language restrictions. Additional sources were identified through manual screening of reference lists and citation tracking.

The search strategy combined controlled vocabulary (MeSH terms) and free-text keywords related to “Enterococcus faecalis”, “intracanal medicaments”, “calcium hydroxide”, “triple antibiotic paste”, “biofilm”, and “antimicrobial resistance”. Boolean operators (AND/OR) were applied to optimize sensitivity and specificity. The complete search strategies for each database are provided in Supplementary Table S1.

### Selection process

Two reviewers independently screened titles and abstracts to identify potentially eligible studies. Full-text articles were subsequently assessed for inclusion based on predefined criteria. Discrepancies were resolved through discussion, and when consensus was not reached, a third reviewer acted as arbitrator.

### Data collection process

Data extraction was conducted independently by two reviewers using a standardized and pilot-tested extraction form. Extracted data included bibliographic details, study design, experimental model, characteristics of the microbial inoculum, type and concentration of intracanal medicament, exposure time, and reported outcomes.

Any disagreements during data extraction were resolved through consensus.

### Data items

The following variables were collected: study characteristics (authors, year, country), experimental conditions (biofilm or planktonic models, dentin depth, incubation period), type of medicament, outcome measures (CFU, MIC, MBC, log reduction), and indicators of antimicrobial persistence or resistance.

### Risk of bias assessment

Risk of bias was evaluated using design-specific tools. For clinical studies, the Cochrane Risk of Bias 2 (RoB 2) tool was applied (10).

For *in vitro* and *ex vivo* studies, an adapted quality assessment framework was developed to ensure methodological transparency and consistency across experimental designs. Because the review was expected to include laboratory-based evidence, this framework was designed to evaluate methodological features that are not fully addressed by conventional clinical risk-of-bias tools, including specimen standardization, allocation of experimental units, reproducibility of contamination and biofilm models, consistency of experimental procedures, and objectivity of microbiological outcome assessment. This framework was informed by established risk of bias principles and tailored to laboratory-based research, focusing on methodological rigor, reproducibility, and clinical relevance.

The assessment included four domains: (1) selection bias (e.g., sample allocation procedures and baseline standardization), (2) performance bias (e.g., operator consistency, protocol standardization, and use of blinding where applicable), (3) outcome measurement bias (e.g., objectivity, validity, and reproducibility of microbiological assays such as CFU, MIC, or MBC), and (4) reporting bias (e.g., completeness and transparency of outcome reporting).

For the adapted assessment of *in vitro* and *ex vivo* studies, explicit criteria were applied to assign judgments within each domain. A judgment of low concern was assigned when the study clearly reported appropriate methodological safeguards for the domain being assessed. For selection bias, this included standardized specimen preparation, comparable baseline conditions, and random or clearly balanced allocation of samples to experimental groups. For performance bias, low concern required consistent application of the experimental protocol across groups, adequate description of contamination, incubation, exposure, and medication procedures, and, when applicable, blinding or other measures to reduce operator-related variability. For outcome measurement bias, low concern was assigned when objective and reproducible antimicrobial outcomes were used, with sufficient methodological detail. For reporting bias, low concern required complete reporting of the prespecified antimicrobial outcomes and sufficient numerical or descriptive information to support interpretation.

A judgment of some concerns was assigned when the study used an apparently appropriate experimental design and objective antimicrobial outcomes but provided incomplete information on one or more methodological safeguards, such as randomization, allocation procedures, operator consistency, blinding, protocol standardization, or completeness of outcome reporting. This category was used conservatively when the available information was insufficient to confirm low concern but did not indicate a clear flaw likely to invalidate the findings.

A judgment of high concern was assigned when there were explicit methodological limitations likely to compromise internal validity, reproducibility, or clinical interpretability. These included poorly representative experimental models for intracanal medication, absence of appropriate controls, major imbalance or lack of standardization between groups, unclear or potentially biased outcome measurement, inadequate description of antimicrobial testing procedures, or incomplete reporting of outcomes that prevented meaningful interpretation.

Overall judgments were derived from the domain-level assessments. Studies were classified as having low overall concern only when all or nearly all domains were judged as low concern and no major limitation was identified. Studies were classified as having some overall concerns when one or more domains had incomplete reporting but no clear critical limitation. Studies were classified as having high overall concern when at least one domain presented a serious limitation likely to affect the reliability or reproducibility of the findings.

When methodological details were insufficiently reported, the corresponding domain was conservatively classified as “some concerns” rather than assuming adequate conduct. Assessments were performed independently by two reviewers using these predefined criteria, and disagreements were resolved through discussion and consensus.

### Certainty of evidence (GRADE assessment)

The certainty of evidence for clinical studies was evaluated using the GRADE framework (11), considering risk of bias, inconsistency, indirectness, imprecision, and publication bias.

For *in vitro* and *ex vivo* studies, an adapted certainty-of-evidence approach was applied to account for the specific characteristics of laboratory-based endodontic research. This approach was informed by GRADE principles but tailored to experimental designs in which conventional certainty domains, such as imprecision and publication bias, are not always directly applicable in the same way as in clinical intervention studies. The following domains were considered: (1) methodological quality, derived from the adapted risk-of-bias assessment; (2) consistency of findings across studies evaluating similar medicaments or antimicrobial strategies; (3) magnitude of antimicrobial effect, based on reported microbiological outcomes such as CFU reduction, log reduction, MIC, MBC, or residual antibacterial activity; and (4) clinical relevance, defined as the extent to which the experimental model approximated intracanal conditions, including the use of dentin specimens, biofilm models, dentinal tubule penetration, *ex vivo* models, or clinical root canal settings.

Evidence from *in vitro* and *ex vivo* studies was initially considered as low certainty, given the inherent indirectness of laboratory-based models in relation to clinical endodontic decision-making. The certainty could be downgraded when there were important methodological concerns, inconsistent findings across comparable studies, limited or unclear antimicrobial effects, simplified models with low clinical approximation, incomplete reporting of key experimental procedures, or outcome measures that were difficult to compare or interpret. Evidence could be downgraded to very low certainty when several of these limitations were present simultaneously or when the findings were based on weak experimental models with poor reproducibility or limited interpretability.

The certainty could be maintained as low when antimicrobial effects were reported but the evidence remained limited by laboratory indirectness, incomplete reporting, or restricted comparability across studies. The certainty could be upgraded to moderate when studies showed consistent antimicrobial effects, used objective and reproducible microbiological outcomes, presented acceptable methodological quality, and employed models with at least partial clinical relevance, such as dentin blocks, tooth specimens, mature biofilm models, or residual antibacterial-effect assessments. For laboratory-based evidence, including *in vitro* and *ex vivo* studies, the highest rating was interpreted cautiously because these models remain indirect in relation to clinical effectiveness. Evidence from well-conducted *ex vivo* studies with low methodological concern, objective outcome measurement, adequate reporting, and clinically relevant features such as infected dentin models, standardized contamination, depth-specific sampling, and blinded outcome assessment could be upgraded to moderate certainty within the experimental or translational context, but not interpreted as high certainty of clinical effectiveness.

The final certainty rating was therefore based on the combined judgment across methodological quality, consistency, magnitude of effect, and clinical relevance. These ratings should be interpreted as certainty within the experimental or translational context of the included evidence, rather than as direct certainty for clinical effectiveness in patients. Final certainty ratings were categorized as high, moderate, low, or very low. Assessments were performed independently by two reviewers, and disagreements were resolved through discussion and consensus.

### Data synthesis and statistical analysis

A structured domain-based interpretative synthesis was used to organize heterogeneous evidence when direct quantitative pooling was limited by differences in study design, antimicrobial models, medicament formulations, exposure times, and outcome metrics. This approach was not intended to represent a distinct or novel meta-analytic method and did not generate a pooled numerical estimate for all comparisons. Instead, it provided a transparent interpretative structure for grouping the evidence according to predefined domains that reflect the biological and translational performance of intracanal medicaments against *E. faecalis*.

The five domains were selected *a priori* based on their relevance to endodontic antimicrobial performance: antimicrobial effectiveness, biofilm disruption capacity, dentinal penetration, persistence of antimicrobial effect, and clinical relevance. Antimicrobial effectiveness was evaluated primarily from quantitative microbiological outcomes such as CFU reduction, log reduction, MIC, and MBC. Biofilm disruption capacity was considered when studies used mature biofilm models, microscopic assessment, or biofilm-associated bacterial reduction. Dentinal penetration was assessed when antimicrobial activity was reported at specific dentinal depths or within dentin tubule models. Persistence of antimicrobial effect was considered when residual antibacterial activity, reinoculation, or sustained antimicrobial action was evaluated. Clinical relevance was judged according to the extent to which the study model approximated clinical intracanal conditions, with greater weight given to *in vivo* and *ex vivo* models than to simplified planktonic or agar-based assays.

The evaluation within each domain was descriptive and comparative rather than based on a formal numerical scoring system. Medicament performance was interpreted according to predefined qualitative criteria, including the magnitude of the reported antimicrobial effect, consistency of findings across studies, use of clinically relevant models, and methodological limitations identified during risk-of-bias assessment. Therefore, classifications such as high, moderate, variable, or limited performance should be understood as structured interpretative judgments derived from reported study outcomes, not as subjective impressions or independently validated quantitative scores.

When quantitative pooling was considered appropriate, effect sizes were calculated as standardized mean differences using Hedges' g. Random-effects models were planned because methodological and clinical heterogeneity was expected across studies. For exploratory quantitative syntheses with a limited number of directly comparable studies, a DerSimonian–Laird random-effects model was used to estimate the pooled effect. Heterogeneity was assessed using the Q statistic, I² statistic, and tau² estimates. Quantitative findings were interpreted cautiously when the number of studies was small, outcome metrics differed, or substantial heterogeneity was detected.

All statistical analyses were conducted using Python (version 3.11), with appropriate scientific libraries. Forest plots were generated to visualize individual and pooled effect estimates. Statistical significance was set at *p* < 0.05.

## Results

### Study selection process

**Figure 1:**
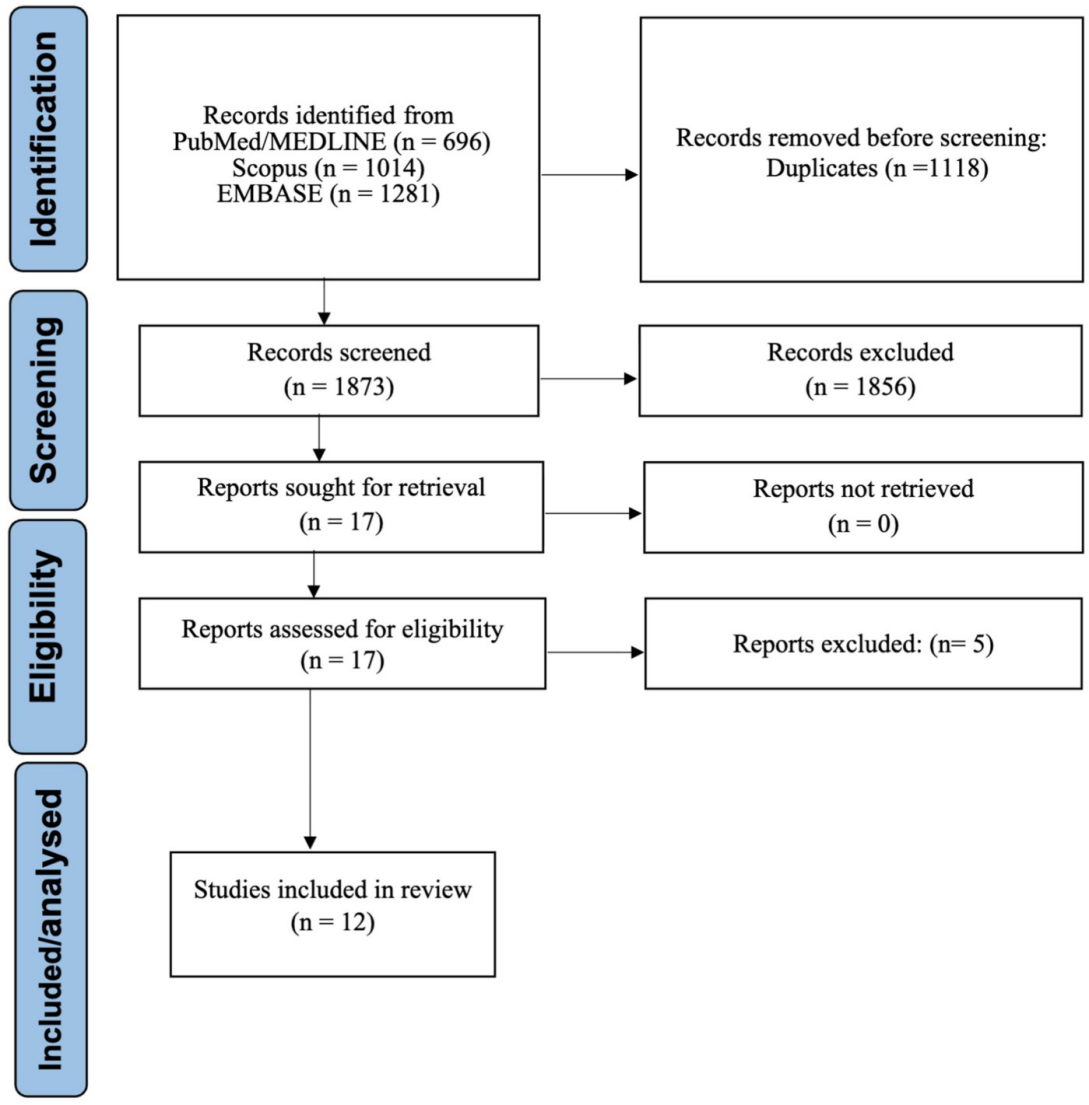
PRISMA flow diagram.

The study selection process is summarized in the PRISMA flow diagram (Figure 1). A total of 2,911 records were initially identified through database searching across PubMed/MEDLINE, Scopus, and Embase. After the removal of duplicate records, 1,873 studies remained for title and abstract screening. During this phase, records were excluded for not meeting the predefined eligibility criteria, primarily because they did not evaluate intracanal medicaments, did not focus on *E. faecalis* as the primary microorganism, or did not report relevant quantitative antimicrobial outcomes.

A total of 17 articles were subsequently assessed in full text. Following detailed evaluation, five studies were excluded due to reasons such as the absence of quantitative microbiological outcomes, exclusive focus on irrigants or non-medication interventions, lack of species-specific data for *E. faecalis*, or failure to meet other predefined inclusion criteria. The specific reasons for the exclusion of these studies are detailed in Supplementary Table S2. Ultimately, 12 studies met all eligibility criteria and were included in the qualitative synthesis (12–23).

### Descriptive characteristics of included studies

The 12 included studies showed substantial methodological diversity but remained aligned with the review objective (Table 1). Ten investigations were laboratory-based, comprising predominantly *in vitro* designs (13, 14, 16, 18–22) and two *ex vivo* studies (15, 17), whereas two were *in vivo* clinical studies performed in patients requiring endodontic treatment (12, 23). Geographically, most studies were conducted in India (12–14, 16, 18–21, 23), two were performed in Iran (17, 22), and one was undertaken across institutions in Indonesia and the United Arab Emirates (15). Sample models included extracted human teeth, dentin blocks or cylinders, root canal segments, and clinical root canals (12–23).

**Table 1:** Descriptive characteristics of the included studies.

Authors (Year), Country	Study design	Sample type	Sample size	Incubation/assessment period	Medicaments evaluated	Outcome measures
Nikhade et al. India (12)	*In vivo* clinical study	Mandibular premolars with necrotic or infected pulp	30 teeth/patients (15 per group)	Assessment before instrumentation, after instrumentation, and after 7 days of intracanal medication	Triple antibiotic paste (TAP), bromelain paste	CFU/mL
Halkai et al. India (13)	*In vitro* study	Root dentin blocks from extracted human single-rooted teeth	30 dentin blocks (10 per group)	Two-week biofilm model; 24 h treatment period	Biosynthesized silver nanoparticles, 2% chlorhexidine, sterile distilled water control	MIC; CFU/mL
Babu et al. India (14)	*In vitro* study	Extracted mandibular premolars	93 teeth total	Assessment at 24 h, 7 days, and 14 days	No medicament control, calcium hydroxide, calcium hydroxide plus omeprazole	CFU
Sidiqa et al. Indonesia/United Arab Emirates (15)	*Ex vivo* study	Root canal specimens from extracted single-rooted mandibular premolars	27 specimens (9 per group)	Twenty-one-day infection period; 7-day intracanal medication	Calcium hydroxide nanoparticles with nisin, calcium hydroxide nanoparticles alone, untreated control	MIC50; FIC; CFU at different dentinal depths; relative gene expression
Harshitha et al. India (16)	*In vitro* study	Decoronated single-rooted premolars	120 teeth	Twenty-one-day infection period; evaluation at day 1 and day 7	Nisin, calcium hydroxide, and triple antibiotic paste, each combined with distilled water or 2% chitosan	CFU
Ahangari et al. Iran (17)	*Ex vivo* study	Dentin cylinders from extracted single-rooted teeth with dual-species biofilm	56 teeth total (10 per group plus 6 for SEM validation)	Twenty-one-day biofilm formation; 7-day exposure to medicaments	Propolis, calcium hydroxide, triple antibiotic paste, modified triple antibiotic paste, no-medicament control	log10 CFU/mg at 200 and 400 µm; microorganism-free specimens; SEM validation
Valan et al. India (18)	*In vitro* study	Agar diffusion, broth microdilution, and hydrogel characterization model	Triplicate microbiological assays	Antibacterial assessment after incubation; MIC/MBC determined after broth microdilution	TAP, TAP hydrogel, modified TAP, modified TAP hydrogel, plain hydrogel	Inhibition zones; MIC; MBC; SEM-based hydrogel characterization
Mekala et al. India (19)	*In vitro* study	Extracted single-rooted permanent teeth	40 teeth (10 per group)	Seven-day infection period; 7-day medicament application	Ampicillin plus ceftriaxone, diclofenac sodium, modified triple antibiotic paste, calcium hydroxide	MIC; CFU (before/after medication); confocal laser microscopy
Mandal et al. India (20)	*In vitro* study	Tooth blocks from extracted single-rooted human teeth	80 tooth blocks	Twenty-one-day infection period; 21-day medication period	TAP with 2% chlorhexidine, TAP with propylene glycol, TAP with normal saline, positive control, negative control	CFU/mL
Punathil et al. India (21)	*In vitro* study	Middle root segments from permanent maxillary central incisors	40 specimens (10 per group)	Five-day infection period; 1-week medicament application	Calcium hydroxide with 2% chlorhexidine, calcium hydroxide with 5% povidone-iodine, calcium hydroxide with saline, control	CFU/mg dentin; pH
Afkhami et al. Iran (22)	*In vitro* study	Standardized human teeth/root dentin model	70 teeth (6 experimental groups of 10; 2 control groups of 5)	One-week drug therapy followed by 24 h reinoculation	2% chlorhexidine gel, calcium hydroxide, calcium hydroxide/chlorhexidine, triple antibiotic paste, double antibiotic paste, calcium hydroxide/silver nanoparticles, controls	Residual antibacterial effect based on CFU/mL
Bhupal et al. India (23)	Randomized *in vivo* clinical study	Single-rooted teeth with necrotic pulp and periapical pathology	24 patients/teeth (8 per group)	Microbiological sampling before instrumentation and after 7 days of intracanal medication	TAP, 2% chlorhexidine gel, bromelain paste	CFU/mL

CFU, colony-forming units; FIC, fractional inhibitory concentration; MIC, minimum inhibitory concentration; MBC, minimum bactericidal concentration; SEM, scanning electron microscopy; TAP, triple antibiotic paste.

Calcium hydroxide, either alone or in combination with other agents, was the most frequently studied medication (14–17, 19, 21, 22). Antibiotic-based formulations were also recurrent, including TAP, double antibiotic paste (DAP), modified TAP, and hydrogel-based antibiotic systems (12, 16–20, 22, 23). Bhupal et al. (23) evaluated and compared the TAP, 2% chlorhexidine gel, and bromelain paste in a randomized clinical setting, using quantitative microbiological assessment based on colony-forming units per milliliter (CFU/mL) of *E. faecalis*.

Several studies evaluated approaches intended to improve antimicrobial performance in clinically challenging environments, such as nanoparticle-based formulations, alternative vehicles, chitosan-containing preparations, bromelain paste, omeprazole combinations, propolis, and nisin-containing systems (12–19, 21–23). Outcomes were mainly quantitative and microbiological, most commonly CFU counts or derived CFU-based measures, while some studies additionally reported MIC, MBC, checkerboard/fractional inhibitory concentration (FIC) data, confocal or scanning electron microscopy findings, or gene expression analysis (13–19, 21, 22). This heterogeneity in experimental models, medication formulations, and outcome reporting supports the use of a structured domain-based interpretative synthesis rather than a single uniform quantitative comparison across all included studies.

### Additional experimental characteristics

A more detailed examination of the experimental conditions revealed variability and, in some cases, incomplete reporting across the included studies. Most laboratory-based investigations employed *E. faecalis* biofilm models; however, explicit characterization of the microbial inoculum, including strain identification or biofilm maturation protocols, was not consistently detailed across all studies. In contrast, the two *in vivo* clinical studies (12, 23) evaluated naturally occurring polymicrobial infections, although microbiological outcomes were reported specifically for *E. faecalis* using culture-based quantification methods.

Only a limited number of studies incorporated polymicrobial conditions under controlled experimental settings, while the majority relied on mono-species models, which may not fully reflect the clinical complexity of root canal infections (17). This distinction is particularly relevant when comparing laboratory findings with clinical evidence, such as that provided by Bhupal et al. (23), where bacterial samples were collected from infected root canals and analyzed for *E. faecalis* counts (CFU/mL) before and after intracanal medication.

Information regarding dentinal penetration depth and antimicrobial activity at different levels of the root canal system was reported in only a few studies, with some assessing specific depths such as 200–400 µm, whereas others did not provide this level of detail (17, 21). Such parameters are inherently difficult to standardize or assess in clinical studies, including Bhupal et al. (23), where microbial sampling was performed using paper points rather than depth-specific dentin analysis.

Although all studies described the type of intracanal medication evaluated, reporting of concentrations, formulations, or dosage parameters was variable and not consistently comparable across studies (12–23). In Bhupal et al. (23), the composition of the medicaments was clearly defined, including TAP, 2% chlorhexidine gel, and bromelain paste.

Exposure times also differed considerably, ranging from short-term assessments (e.g., 24 h) to longer periods of up to 21 days, reflecting heterogeneity in experimental design (13, 14, 20). In the clinical studies (12, 23), intracanal medicaments were typically evaluated after a 7-day application period, aligning with common endodontic practice.

Finally, indicators of antimicrobial persistence or residual antibacterial effects were evaluated in a limited number of studies, despite their clinical relevance for preventing reinfection. Overall, this variability in experimental conditions and reporting limits direct comparability between studies and should be considered when interpreting the findings of this review.

### Risk of bias assessment

The risk of bias varied across the included studies (Table 2). The two clinical studies (12, 23), assessed using RoB 2, were judged as presenting some concerns overall. In both cases, random allocation was reported; however, allocation concealment and blinding procedures were not described in sufficient detail. In Bhupal et al. (23), although the study was described as randomized and used objective microbiological outcomes based on CFU/mL of *E. faecalis*, insufficient information regarding allocation concealment and masking of operators or outcome assessors led to a judgment of some concerns.

**Table 2:** Risk of bias assessment of the included studies.

Study	Tool	Selection bias	Performance bias	Outcome measurement	Reporting bias	Overall judgment	Main reason
Nikhade et al. (12)	RoB 2	Some concerns	Some concerns	Low	Some concerns	Some concerns	Limited concealment/blinding details
Halkai et al. (13)	Adapted	Some concerns	Some concerns	Low	Some concerns	Some concerns	No explicit randomization/blinding
Babu et al. (14)	Adapted	Some concerns	Some concerns	Low	Some concerns	Some concerns	Allocation/blinding unclear
Sidiqa et al. (15)	Adapted	Some concerns	Some concerns	Low	Some concerns	Some concerns	Blinding not detailed
Harshitha et al. (16)	Adapted	Some concerns	Some concerns	Low	Some concerns	Some concerns	Randomization not explicit
Ahangari et al. (17)	Adapted	Low	Low	Low	Low	Low	Randomization and blinding reported
Valan et al. (18)	Adapted	High	High	Some concerns	Some concerns	High	Weak model and no blinding
Mekala et al. (19)	Adapted	Some concerns	Some concerns	Low	Some concerns	Some concerns	No blinding reported
Mandal et al. (20)	Adapted	Some concerns	Some concerns	Low	Some concerns	Some concerns	Randomization unclear
Punathil et al. (21)	Adapted	Some concerns	Some concerns	Low	Some concerns	Some concerns	Blinding not specified
Afkhami et al. (22)	Adapted	Some concerns	Some concerns	Low	Some concerns	Some concerns	No explicit blinding
Bhupal et al. (23)	RoB 2	Some concerns	Some concerns	Low	Some concerns	Some concerns	Randomization reported; allocation concealment and blinding not detailed

Low concern indicated that the methodological safeguards for the assessed domain were clearly reported and appropriate. Some concerns indicated incomplete reporting or uncertainty regarding one or more safeguards, without evidence of a major flaw. High concern indicated an explicit limitation likely to affect internal validity, reproducibility, or clinical interpretability. For laboratory-based studies, incomplete reporting of randomization, blinding, allocation procedures, or protocol standardization was conservatively classified as some concerns unless a clear methodological flaw justified a high-concern judgment.

Among the predominantly *in vitro* studies (13, 14, 16, 18–22) and the two *ex vivo* studies (15, 17), most were judged as having some overall concerns. This judgment mainly reflected incomplete reporting rather than clear evidence of serious methodological flaws. The most frequent sources of uncertainty were insufficient description of allocation procedures, absence of explicit operator or assessor blinding, and limited detail regarding protocol standardization. Outcome measurement was generally judged at low concern because antimicrobial activity was assessed using objective and reproducible endpoints such as CFU counts, log reduction, MIC, MBC, and other standardized microbiological assays.

Only one *ex vivo* study (17) clearly reported both random allocation and blinding of the operator and outcome assessor and was therefore judged as having low concerns overall. One *in vitro* study (18), which relied mainly on agar diffusion and hydrogel characterization, with limited representation of intracanal conditions, was judged as presenting high concerns overall. No study was excluded on the basis of risk of bias alone.

### Structured domain-based interpretative synthesis of antimicrobial effectiveness

**Figure 2:**
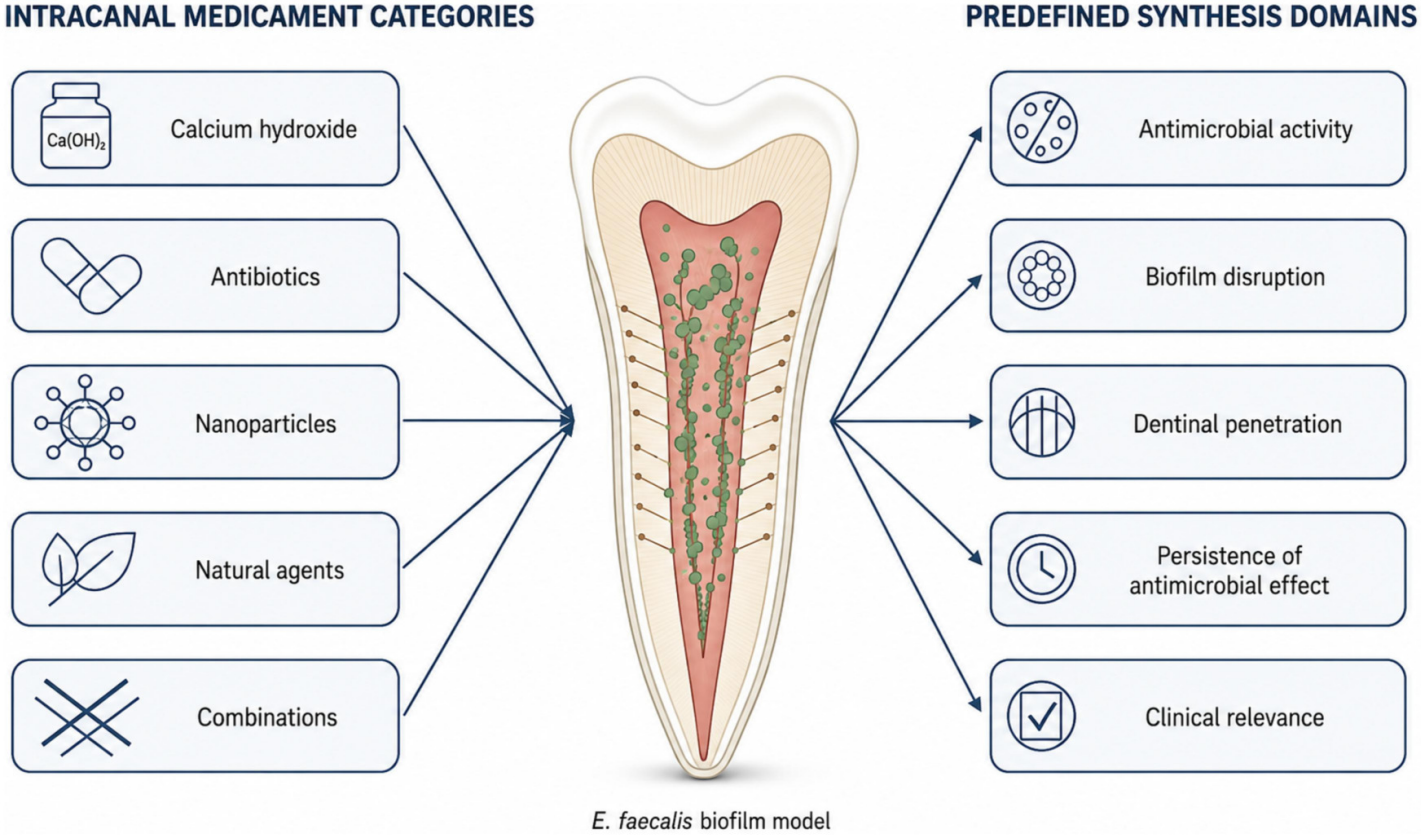
Conceptual schematic of the predefined domains used in the structured domain-based interpretative synthesis. The figure illustrates the main intracanal medicament categories and the predefined domains used to organize the synthesis, including antimicrobial activity, biofilm disruption, dentinal penetration, persistence of antimicrobial effect, and clinical relevance. The central schematic represents a cross-sectional view of an infected tooth, highlighting *E. faecalis* biofilm colonization and dentinal tubule penetration. The figure is intended solely as a conceptual illustration of the synthesis framework and does not represent strength of evidence, treatment ranking, comparative superiority, certainty ratings, or a quantitative scoring system.

The structured domain-based interpretative synthesis organized heterogeneous evidence using the five predefined domains described in the Methods section: antimicrobial effectiveness, biofilm disruption capacity, dentinal penetration, persistence of antimicrobial effect, and clinical relevance. These domains were selected because they capture complementary aspects of intracanal medicament performance against *E. faecalis*, ranging from direct bacterial reduction to activity within biofilm and dentin environments and the translational applicability of the experimental model. The domain-based interpretation was based on reported quantitative and qualitative outcomes, including CFU reduction, log reduction, MIC/MBC values, biofilm-related findings, dentinal depth assessments, residual antibacterial effects, and model proximity to clinical conditions. Thus, the structured domain-based interpretative synthesis was intended to organize the evidence for interpretative comparison rather than to provide a formal numerical ranking of medicaments. The predefined domains used to organize the synthesis are illustrated conceptually in Figure 2.

Calcium hydroxide, either alone or combined with adjunctive agents, was one of the most frequently evaluated intracanal medicaments across the included studies (14–17, 19, 21, 22). Overall, calcium hydroxide alone demonstrated moderate antimicrobial activity against *E. faecalis*, although its effectiveness was generally lower than that observed for antibiotic-based formulations. Several investigations reported enhanced antimicrobial effects when calcium hydroxide was combined with adjunctive agents such as chlorhexidine, silver nanoparticles, or omeprazole, suggesting a potential synergistic interaction that improves antimicrobial performance (14, 19, 21, 22). However, findings were not entirely consistent, particularly in studies assessing deeper dentinal layers, where persistent bacterial viability was reported, indicating limited penetration capacity under complex anatomical conditions (17, 21).

Antibiotic-based medicaments, particularly TAP and its modified formulations, consistently demonstrated high antimicrobial effectiveness across most included studies (12, 16–20, 22, 23). These formulations were associated with greater reductions in CFU counts, lower MIC and MBC values, and improved biofilm disruption. In the randomized clinical study by Bhupal et al. (23), bromelain paste showed the greatest reduction in *E. faecalis* CFU/mL, followed by TAP and 2% chlorhexidine gel, highlighting the potential of alternative bioactive agents in clinical settings. Some studies further explored modified delivery systems, including hydrogel-based formulations and alternative vehicles, aiming to enhance drug release and reduce cytotoxicity while maintaining antimicrobial efficacy (18, 20). Despite these favorable findings, variability in formulation composition, concentration, and application protocols limits direct comparability between studies.

Several studies also explored alternative or adjunctive antimicrobial strategies, including nisin-based formulations, propolis, chitosan combinations, bromelain paste, and nanoparticle-enhanced medicaments (12, 15–17, 19, 21, 22). These approaches demonstrated promising antimicrobial activity, in some cases comparable to conventional medicaments. Notably, nanoparticle-based systems and bioactive peptides were associated with improved penetration and biofilm disruption in experimental models (13, 15, 17). However, these findings were derived from a limited number of studies with heterogeneous methodologies, and their reproducibility and clinical applicability remain uncertain.

Substantial variability in experimental design was observed across the included studies and appears to have influenced antimicrobial outcomes. Differences were noted in exposure time, ranging from short-term assessments (24 h) to extended periods of up to 21 days (13, 14, 20), as well as in biofilm maturation protocols, depth of bacterial penetration within dentinal tubules, and the use of mono-species vs. polymicrobial models, the latter being only rarely represented (17). Only a limited number of studies evaluated antimicrobial activity at different dentinal depths or assessed residual antibacterial effects following reinoculation, despite their clinical relevance (17, 22). This variability precludes direct quantitative comparison and underscores the need for cautious interpretation of antimicrobial effectiveness.

Despite this heterogeneity, several consistent patterns emerged across the evidence base. Antibiotic-based medicaments demonstrated the highest antimicrobial effectiveness across most studies (12, 16–20, 22, 23), whereas calcium hydroxide alone showed more variable and generally lower effectiveness, particularly in deeper dentinal regions (17, 21). Combination therapies tended to enhance antimicrobial performance, suggesting potential synergistic interactions (14, 19, 22), while novel agents showed promising results but limited reproducibility and insufficient evidence for clinical translation (13, 15, 17). The inclusion of clinical evidence from Bhupal et al. (23) highlights the variability of antimicrobial effectiveness in clinical settings and suggests that alternative agents such as bromelain may achieve comparable or greater bacterial reduction than conventional antibiotic-based formulations.

### Exploratory meta-analysis

A direct quantitative comparison was only feasible for calcium hydroxide vs. TAP. After full-text assessment, only two studies provided sufficiently comparable data for inclusion in this exploratory synthesis (17, 22). Although Punathil et al. (21) met the general eligibility criteria, it did not include a TAP arm and was therefore not eligible for this specific comparison.

Afkhami et al. (22) reported a substantial reduction in bacterial load (CFU/mL) in the TAP group compared with calcium hydroxide, resulting in a very large standardized effect size (Hedges' *g* = 10.10; 95% CI: 6.69 to 13.52). In contrast, Ahangari et al. (17), using log₁₀ CFU/mg at a dentinal depth of 400 μm, observed no significant difference between groups (Hedges' *g* = −0.31; 95% CI: −1.19 to 0.58).

Given the limited number of directly comparable studies, a DerSimonian–Laird random-effects model was applied only as an exploratory approach. The pooled effect estimate was large but highly imprecise (Hedges' *g* = 4.76; 95% CI: −5.43 to 14.96), with extreme heterogeneity (*Q* = 33.46, df = 1; I² = 97.0%; *τ*² = 52.55).

The marked inconsistency between studies, both in magnitude and direction of effect, appears to reflect substantial methodological heterogeneity, including differences in outcome metrics (CFU/mL vs. log₁₀ CFU/mg), sampling depth, and experimental conditions. Consequently, the pooled estimate should be interpreted with caution and should not be considered a definitive quantitative summary.

**Figure 3:**
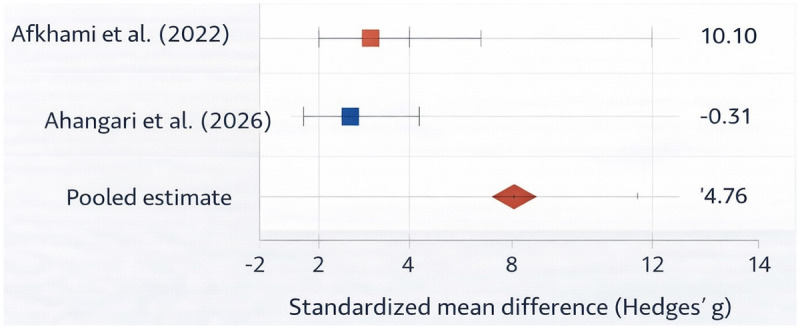
Exploratory forest plot of calcium hydroxide vs. triple antibiotic paste. Forest plot showing the standardized mean differences (Hedges’ g) for the direct comparison between calcium hydroxide and triple antibiotic paste based on the two eligible studies (17, 22). The estimate from Ahangari et al. (17) corresponds to measurements obtained at a dentinal depth of 400 μm. The pooled effect was calculated using a DerSimonian–Laird random-effects model. The wide confidence interval and substantial heterogeneity (I² = 97.0%) indicate that the combined estimate should be interpreted with caution and considered exploratory.

Overall, these findings indicate that quantitative synthesis is severely constrained by limited direct comparability, the inclusion of only two studies, and extreme heterogeneity (Figure 3). Therefore, the pooled estimate should be regarded as hypothesis-generating rather than confirmatory. The exploratory meta-analysis was used only to illustrate the limited direct comparability of the available data and should not be interpreted as establishing the superiority of one intracanal medicament over another. Accordingly, the structured domain-based interpretative synthesis remains the primary approach for organizing and interpreting the heterogeneous evidence in this review.

### Certainty of evidence

The certainty of evidence across the included studies was predominantly low to moderate (Table 3). The two clinical studies (12, 23) were rated as low certainty due to concerns related to risk of bias and imprecision. Although objective microbiological outcomes were reported in both studies, limited detail regarding allocation concealment and blinding reduced confidence in the estimates. In Bhupal et al. (23), despite the randomized design and the clear demonstration of greater CFU/mL reduction with bromelain, followed by TAP and 2% chlorhexidine gel after a 7-day intracanal application, the certainty of evidence was downgraded due to risk of bias and imprecision.

**Table 3:** Certainty of evidence assessment of the included studies.

Study	Design	Methodological quality	Consistency	Clinical relevance	Magnitude	Overall certainty
Nikhade et al. (12)	*In vivo* clinical study	Some concerns	Consistent	High	Moderate	Low
Halkai et al. (13)	*In vitro*	Some concerns	Consistent	Moderate	Moderate	Moderate
Babu et al. (14)	*In vitro*	Some concerns	Consistent	Low	Moderate	Moderate
Sidiqa et al. (15)	*Ex vivo*	Some concerns	Consistent	Moderate	Moderate	Moderate
Harshitha et al. (16)	*In vitro*	Some concerns	Consistent	Moderate	Moderate	Moderate
Ahangari et al. (17)	*Ex vivo*	Low	Consistent	Moderate	High	Moderate
Valan et al. (18)	*In vitro*	High	Variable	Low	Low	Low
Mekala et al. (19)	*In vitro*	Some concerns	Consistent	Low	Moderate	Moderate
Mandal et al. (20)	*In vitro*	Some concerns	Consistent	Low	Moderate	Moderate
Punathil et al. (21)	*In vitro*	Some concerns	Consistent	Moderate	Moderate	Moderate
Afkhami et al. (22)	*In vitro*	Some concerns	Consistent	Moderate	Moderate	Moderate
Bhupal et al. (23)	Randomized *in vivo* clinical study	Some concerns	Consistent	High	Moderate–High	Low

For *in vitro* and *ex vivo* studies, certainty ratings were based on an adapted framework informed by GRADE principles. Evidence was initially considered low certainty because of laboratory indirectness. Ratings were downgraded for important methodological concerns, inconsistency, limited or unclear antimicrobial effect, low clinical approximation, incomplete reporting, or poor comparability of outcomes. Ratings were upgraded when findings were consistent, effects were large or biologically meaningful, outcomes were objective and reproducible, and the model approximated clinical intracanal conditions. For laboratory-based evidence, including *in vitro* and *ex vivo* studies, certainty ratings refer only to the experimental or translational context. Even when methodological quality was high and antimicrobial effects were consistent, *ex vivo* evidence was not interpreted as high certainty for clinical effectiveness because of residual indirectness.

Among the *in vitro* studies (13, 14, 16, 18–22), the certainty of evidence was generally low to moderate. These studies demonstrated antimicrobial activity under controlled laboratory conditions; however, their certainty was frequently downgraded due to indirectness, as experimental models do not fully replicate the clinical intracanal environment, and due to incomplete reporting of methodological safeguards in some cases.

The two *ex vivo* studies (15, 17) provided greater clinical approximation than purely *in vitro* systems and were therefore considered less indirect. However, because *ex vivo* evidence remains laboratory-based and does not directly establish clinical effectiveness, neither study was interpreted as providing high certainty. Ahangari et al. (17) showed higher methodological rigor and was therefore rated as moderate certainty within the experimental or translational context, whereas Sidiqa et al. (15) was also rated as moderate certainty.

Overall, while antimicrobial effects were consistently observed, the certainty of evidence remains limited by indirectness and methodological heterogeneity. The inclusion of clinical evidence from Bhupal et al. (23) illustrates the variability of antimicrobial effects in clinical conditions and partially aligns with experimental findings.

## Discussion

Persistent endodontic infections remain a critical challenge in contemporary endodontics, with *E. faecalis* contributing to post-treatment disease through its ability to survive in harsh environments, penetrate dentinal tubules, and form resistant biofilms (1, 2). This systematic review evaluated the antimicrobial effectiveness of intracanal medicaments by integrating quantitative microbiological outcomes with a structured domain-based interpretative synthesis and, where feasible, exploratory meta-analysis.

Overall, the included studies showed antimicrobial activity against *E. faecalis*, but the magnitude and clinical relevance of these effects varied according to medicament type, experimental model, and methodological design. Antibiotic-based formulations, particularly TAP and its derivatives, generally showed stronger antimicrobial performance across the available evidence (12, 16–20, 22, 23), whereas calcium hydroxide alone showed more variable activity, especially in deeper dentinal regions (17, 21). The clinical study by Bhupal et al. (23) reported greater reductions in *E. faecalis* CFU/mL with bromelain, followed by TAP and 2% chlorhexidine gel. These findings should nevertheless be interpreted cautiously because of methodological heterogeneity and limited direct clinical evidence.

Differences in experimental conditions were a major source of variability across studies. These included differences in biofilm maturation, dentinal penetration depth, exposure time, and microbial models (13–22). Most investigations used mono-species *E. faecalis* biofilms, which do not fully reproduce the polymicrobial nature of clinical endodontic infections (24). In addition, only a few studies assessed antimicrobial activity at specific dentinal depths or evaluated residual antibacterial effects after reinoculation (17, 22), despite the relevance of bacterial persistence in deeper dentinal tubules to treatment failure (25). These methodological differences limit direct comparability and may explain some discrepancies in reported outcomes.

The overall risk of bias across studies was predominantly classified as “some concerns,” reflecting incomplete reporting of key methodological safeguards such as randomization and blinding (13–22). Only one study demonstrated low risk of bias across all domains (17), while one study presented high concerns due to limitations in experimental design and clinical applicability (18). Although outcome measurement was generally robust—given the use of objective microbiological endpoints such as CFU, MIC, and MBC—the lack of transparency in allocation procedures and operator blinding introduces uncertainty regarding internal validity. Similar methodological limitations have been reported in previous reviews of endodontic laboratory studies, highlighting the need for improved reporting standards (26).

The structured domain-based interpretative synthesis helped organize the heterogeneous findings across five predefined domains: antimicrobial effectiveness, biofilm disruption, dentinal penetration, persistence, and clinical relevance. Antibiotic-based medicaments generally showed stronger performance for CFU reduction and biofilm-related outcomes (12, 16–20, 22, 23), consistent with previous reports describing the antimicrobial activity of TAP against *E. faecalis* (5, 27, 28). By contrast, calcium hydroxide alone showed less consistent activity, particularly in deeper dentinal layers (17, 21), which is biologically plausible given the ability of *E. faecalis* to tolerate alkaline stress and persist within biofilm-associated environments (3, 29). Adjunctive formulations, including calcium hydroxide combined with chlorhexidine, silver nanoparticles, or other agents, appeared to improve antimicrobial activity in some models (14, 19, 22). Nanoparticle-enhanced and bioactive agents, including nisin and propolis, also showed promising experimental results (3–32), but the limited and heterogeneous evidence precludes firm clinical interpretation.

The exploratory meta-analysis, limited to two comparable studies (17, 22), showed extreme heterogeneity and inconsistent findings, with one study favoring TAP and the other showing no significant difference. The pooled estimate was therefore highly imprecise and should not be considered clinically definitive. These results support the decision to use the structured domain-based interpretative synthesis as the primary approach for organizing the heterogeneous evidence. Although Bhupal et al. (23) was not eligible for quantitative pooling, its clinical findings suggest that bromelain may achieve substantial antibacterial effects under clinical conditions. Overall, the meta-analysis should be interpreted only as an exploratory complement and not as evidence of comparative superiority.

Higher antimicrobial activity does not necessarily imply greater clinical suitability. Although antibiotic-based medicaments, particularly TAP and modified TAP, showed comparatively strong antibacterial performance against *E. faecalis* (12, 16–20, 22, 23), their use must be balanced against antimicrobial stewardship, potential selection of resistant microorganisms, concentration-dependent cytotoxicity, and tooth discoloration (8, 33–35). Calcium hydroxide showed less consistent activity in dentin and biofilm-associated conditions, but it remains clinically relevant because of its availability, long-standing use, and comparatively favorable biological profile (3, 27, 33). Adjunctive and emerging formulations, including chlorhexidine-containing combinations, nanoparticle-enhanced medicaments, and natural bioactive agents, may help address some limitations of conventional medicaments, but the current evidence remains insufficient to support firm clinical recommendations (13–15, 17, 21–23). Therefore, medicament selection should consider not only bacterial reduction, but also biological safety, antimicrobial stewardship, discoloration risk, ease of use, and the level of clinical evidence supporting each formulation.

The overall certainty of evidence was predominantly low to moderate. The clinical studies were rated as low certainty due to risk of bias and imprecision (12, 23). *In vitro* studies, which constituted the majority of the evidence, were frequently downgraded due to indirectness and variability in experimental conditions (13, 14, 16, 18–22). *Ex vivo* studies provided improved clinical approximation compared with simplified *in vitro* systems, but they remained subject to residual indirectness and were therefore interpreted as providing, at most, moderate certainty within the experimental or translational context (15, 17). These findings are consistent with previous literature emphasizing that laboratory-based evidence, while valuable for mechanistic insights, cannot fully replicate the complexity of the clinical environment (32). Therefore, although antimicrobial effects were consistently observed, confidence in the magnitude and clinical applicability of these effects remains limited.

This systematic review has several strengths. It integrates clinical, *ex vivo*, and *in vitro* evidence; uses a structured domain-based interpretative synthesis to organize heterogeneous findings; and includes an exploratory meta-analysis to transparently show the limited comparability of the available quantitative data. Nevertheless, important limitations remain. Although major biomedical and multidisciplinary databases were searched and reference lists were screened manually, gray literature and additional regional databases were not included. As a result, unpublished studies, conference proceedings, theses, or difficult-to-index laboratory studies may have been missed. In addition, the included studies differed substantially in design, experimental conditions, medicament formulations, and outcome measures. The predominance of *in vitro* evidence is particularly important because these models cannot fully reproduce the complexity of clinical root canal infections, including polymicrobial biofilms, host factors, anatomical variability, medicament diffusion, and treatment-related procedural variables. Therefore, the findings should be interpreted primarily as experimental and translational evidence rather than as direct clinical evidence of comparative effectiveness.

From a clinical perspective, antibiotic-based intracanal medicaments, particularly TAP and its modified formulations, showed comparatively strong antimicrobial performance in the experimental evidence; however, this should not be interpreted as definitive clinical superiority because direct clinical data remain limited. Their use must be balanced against cytotoxicity, antibiotic-resistance concerns, and tooth discoloration (8, 33–35). The available clinical evidence also suggests that bromelain may be a relevant alternative, as one randomized clinical study reported greater reductions in *E. faecalis* CFU/mL than TAP and 2% chlorhexidine gel (23). Calcium hydroxide remains widely used, but its limitations against *E. faecalis* support continued investigation of adjunctive or alternative strategies. Further clinical validation and safety assessment are required before emerging approaches, including nanoparticle-based systems and bioactive agents, can be recommended for routine use.

Previous systematic reviews on antimicrobial strategies against *E. faecalis* have generally focused on specific interventions, including nanoparticle-enhanced calcium hydroxide formulations (36), silver diamine fluoride (37), or calcium hydroxide–chlorhexidine combinations (38). These reviews provide useful intervention-specific evidence, but their narrower scope and limited quantitative comparability restrict broader interpretation across medicament categories.

The present review therefore uses a structured domain-based interpretative synthesis to organize heterogeneous evidence across clinically relevant dimensions, including antimicrobial effectiveness, biofilm-related activity, dentinal penetration, persistence, and clinical relevance. This structured domain-based interpretative synthesis was used as a transparent interpretative framework and should not be considered a validated scoring system or a separate quantitative synthesis method.

Future studies should prioritize well-designed clinical trials with standardized protocols and clinically relevant outcome measures. Greater consistency in experimental models, including the use of polymicrobial biofilms and standardized dentinal depth assessments, would improve comparability across studies. Additionally, future research should focus on evaluating the long-term antimicrobial persistence of medicaments and their effects on treatment outcomes, rather than relying solely on short-term microbiological endpoints. The development of standardized reporting guidelines for *in vitro* and *ex vivo* studies would further enhance methodological quality and reproducibility.

In summary, intracanal medicaments demonstrated antimicrobial activity against *E. faecalis*, but their effects varied according to formulation and experimental conditions. Antibiotic-based medicaments showed comparatively consistent antimicrobial performance, whereas calcium hydroxide alone showed limitations, particularly in deeper dentinal regions. Given the predominance of laboratory-based evidence, substantial heterogeneity, and limited clinical data, these findings should be interpreted cautiously. Further well-designed clinical research is needed to inform evidence-based decision-making in endodontic practice.

## Conclusions

Intracanal medicaments demonstrate consistent antimicrobial activity against *E. faecalis* across clinical, *ex vivo*, and *in vitro* models; however, their effectiveness varies substantially depending on formulation and experimental conditions. Antibiotic-based medicaments, particularly triple antibiotic paste and its modified formulations, showed the most consistent and pronounced antimicrobial effects, especially in terms of biofilm disruption and dentinal penetration. In contrast, calcium hydroxide alone exhibited more variable and generally reduced effectiveness, particularly in deeper dentinal regions, although its performance may be enhanced when combined with adjunctive agents.

Despite these findings, the overall certainty of evidence remains low to moderate due to methodological heterogeneity, indirectness of laboratory-based models, and the limited availability of high-quality clinical data. Because most included studies were *in vitro*, the results should be interpreted primarily as experimental and translational evidence rather than as definitive clinical evidence. The exploratory meta-analysis was based on only two directly comparable studies and showed extreme heterogeneity; therefore, its pooled estimate should be regarded as hypothesis-generating and not as a definitive measure of comparative effectiveness. Further well-designed clinical studies with standardized methodologies are essential to validate these findings and to inform evidence-based selection of intracanal medicaments in endodontic practice.

## Data Availability

The original contributions presented in the study are included in the article/Supplementary Material, further inquiries can be directed to the corresponding author/s.
