# Supplementary material for: Antimicrobial effectiveness of intracanal medicaments against Enterococcus faecalis in endodontics: a systematic review with structured domain-based interpretative synthesis and exploratory meta-analysis
Source: Front Oral Health. 2026 May 29;7:1854046. doi: 10.3389/froh.2026.1854046 (PMC13260536; doi:10.3389/froh.2026.1854046)
Supplement: Supplementary file 1 [file Table1.docx]

Supplementary Table 1. Search Strategy

| Database | Search Strategy |
| --- | --- |
| PubMed (MEDLINE) | ("Enterococcus faecalis"[MeSH Terms] OR "Enterococcus faecalis"[tiab]) AND  ("Root Canal Therapy"[MeSH Terms] OR endodontic*[tiab] OR "root canal"[tiab]) AND  ("Anti-Infective Agents, Local"[MeSH Terms] OR "intracanal medicament*"[tiab] OR  "calcium hydroxide"[tiab] OR chlorhexidine[tiab] OR "triple antibiotic paste"[tiab] OR TAP[tiab]) AND  (biofilm*[tiab] OR dentin[tiab] OR antimicrobial*[tiab] OR CFU[tiab] OR MIC[tiab] OR MBC[tiab]) |
| Scopus | TITLE-ABS-KEY ("Enterococcus faecalis") AND  TITLE-ABS-KEY (endodontic* OR "root canal") AND  TITLE-ABS-KEY ("intracanal medicament*" OR "calcium hydroxide" OR chlorhexidine OR "triple antibiotic paste") AND  TITLE-ABS-KEY (biofilm OR dentin OR antimicrobial OR CFU OR MIC OR MBC) |
| Embase (via Ovid) | ('enterococcus faecalis'/exp OR 'enterococcus faecalis':ti,ab) AND  ('root canal therapy'/exp OR endodontic*:ti,ab OR 'root canal':ti,ab) AND  ('antiinfective agent'/exp OR 'intracanal medicament*':ti,ab OR 'calcium hydroxide':ti,ab OR chlorhexidine:ti,ab OR 'triple antibiotic paste':ti,ab) AND  (biofilm*:ti,ab OR dentin:ti,ab OR antimicrobial*:ti,ab OR cfu:ti,ab OR mic:ti,ab OR mbc:ti,ab) |
