# Supplementary material for: Antimicrobial effectiveness of intracanal medicaments against Enterococcus faecalis in endodontics: a systematic review with structured domain-based interpretative synthesis and exploratory meta-analysis
Source: Front Oral Health. 2026 May 29;7:1854046. doi: 10.3389/froh.2026.1854046 (PMC13260536; doi:10.3389/froh.2026.1854046)
Supplement: Supplementary file 2 [file Table2.docx]

Supplementary Table 2. Excluded Studies and Reasons for Exclusion

| Study | Reason for Exclusion |
| --- | --- |
| Panyakorn et al. (2021) | Did not report quantitative CFU outcomes specific to *E. faecalis*; outcomes were not species-specific. |
| Zancan et al. (2019) | Focused on irrigant protocols rather than intracanal medicaments, not aligned with intervention criteria. |
| Chandwani et al. (2022) | Evaluated antimicrobial effects without reporting CFU/mL or equivalent quantitative microbiological outcomes. |
| Anija et al. (2021) | Lacked species-specific data for *E. faecalis*; results reported for mixed microbiota. |
| Shreya et al. (2021) | Did not meet predefined inclusion criteria due to non-comparable study design and absence of required outcome measures. |
